# Livestock mortality and household food insecurity in Somalia: evidence from the 2020 SDHS

**DOI:** 10.3389/fvets.2026.1889238

**Published:** 2026-07-21

**Authors:** Zakaria Ibrahim Issack

**Affiliations:** 1 One Health Unit, Department of Health Emergencies, Ministry of Health and Human Services, Federal Government of Somalia, Mogadishu, Somalia; 2 Faculty of Health Science, Salaam University, Mogadishu, Somalia; 3 Somherd Solutions, Mogadishu, Somalia

**Keywords:** drought resilience, food insecurity, livestock mortality, pastoralist livelihoods, poultry and small ruminants

## Abstract

**Background:**

Food insecurity remains a major public health challenge in Somalia, where livelihoods depend heavily on livestock production. Recurrent droughts and endemic livestock diseases threaten household resilience, yet national evidence linking species-specific livestock mortality to food insecurity is limited.

**Methods:**

We conducted a cross-sectional analysis of 26,389 households using data from the 2020 Somalia. Demographic and Health Survey (SDHS). Household food insecurity was defined as self-reported insufficient food during the previous 4 weeks. Exposures included drought- and disease-related mortality among camels, cattle, small ruminants, and poultry. Weighted multivariable logistic regression models estimated adjusted odds ratios (aORs) and 95% confidence intervals (CIs), controlling for region, residence type, sex of household head, and household wealth.

**Results:**

Overall, 42.9% of households reported food insecurity. The occurrence of drought-related mortality was most prevalent among small ruminants (91.3%), while disease-related deaths were also most common in this species (22.3%). Drought-related mortality occurrence in cattle (aOR = 1.32; 95% CI: 1.21–1.45) and poultry (aOR = 1.27; 95% CI: 1.09–1.49) was associated with significantly higher odds of food insecurity. Disease-related mortality associations were even stronger, particularly for poultry (aOR = 1.71; 95% CI: 1.45–2.02) and small ruminants (aOR = 1.26; 95% CI: 1.17–1.36). Food insecurity was highest in Gedo, Bakool, and Hiraan regions, while wealthier households had significantly lower risk. Female-headed households were more vulnerable.

**Conclusions:**

Livestock mortality, especially disease-related losses among poultry and small ruminants, is strongly associated with household food insecurity in Somalia. Strengthening veterinary services, disease surveillance, and drought resilience interventions should be prioritized within national food security strategies.

## Introduction

Food insecurity remains a critical global public health challenge, affecting an estimated 673 million people worldwide by 2024 (1). This pervasive issue has profound implications for human physical, social, and cognitive development throughout life, serving as a major disruptor of both individual wellbeing and broader societal stability (2). The consequences

extend beyond immediate hunger, undermining efforts toward sustainable development and poverty alleviation, and perpetuating cycles of malnutrition and disease (3). Achieving food security, defined as ensuring that all people have physical and economic access to sufficient, safe, and nutritious food for an active and healthy life, remains central to multiple Sustainable Development Goals, particularly SDG 2 (zero hunger) (4). In sub-Saharan Africa, food insecurity is disproportionately severe, affecting over 20% of the population in 2024, representing 298.1 million people (3). This region faces unique challenges, characterized by high poverty rates, recurring climate extremes, and heavy reliance on agriculture and livestock for livelihoods (5). Among the most vulnerable populations are pastoral and agropastoral communities, for which livestock represent not merely an economic asset but the backbone of food and income security (6). Pastoralism constitutes the primary livelihood for an estimated 268 million people across Africa's drylands, covering 43% of the continent's landmass, and providing crucial contributions to social, environmental, and economic wellbeing (7). Somalia exemplifies these challenges, ranking among the six countries with “alarming” levels of hunger according to the 2024 Global Hunger Index (6). The country's economy is traditionally agropastoral, with the livestock sector accounting for approximately 40% of the gross domestic product and 65% of the population engaged in livestock-rearing activities. For Somali pastoralists, livestock serves multiple functions: providing milk, meat, and income while representing social status and serving as insurance against economic shocks. However, this heavy dependence on livestock also creates significant vulnerability to climate- and disease-induced livestock mortality (8).

Somalia's livestock population exhibits marked regional and production system variations. Estimates from 2016 to 2017 documented approximately 36.1 million livestock following drought-related losses, comprising an estimated 7.3 million camels, 4.2 million cattle, 20.5 million sheep and goats combined, and approximately 4.1 million poultry (9, 10). Prior to the drought, the national herd exceeded 52.9 million animals (9). These species are distributed differentially across production zones: nomadic populations predominantly keep camels (approximately 71% of camel-owning households are nomadic) and small ruminants (95% of sheep/goat-owning households), whereas urban and peri-urban areas have higher concentrations of poultry (9, 10). Livestock ownership in Somalia is widespread, with 38.6% of all households owning at least one species, rising to 94.8% among nomadic households.

**Figure 1:**
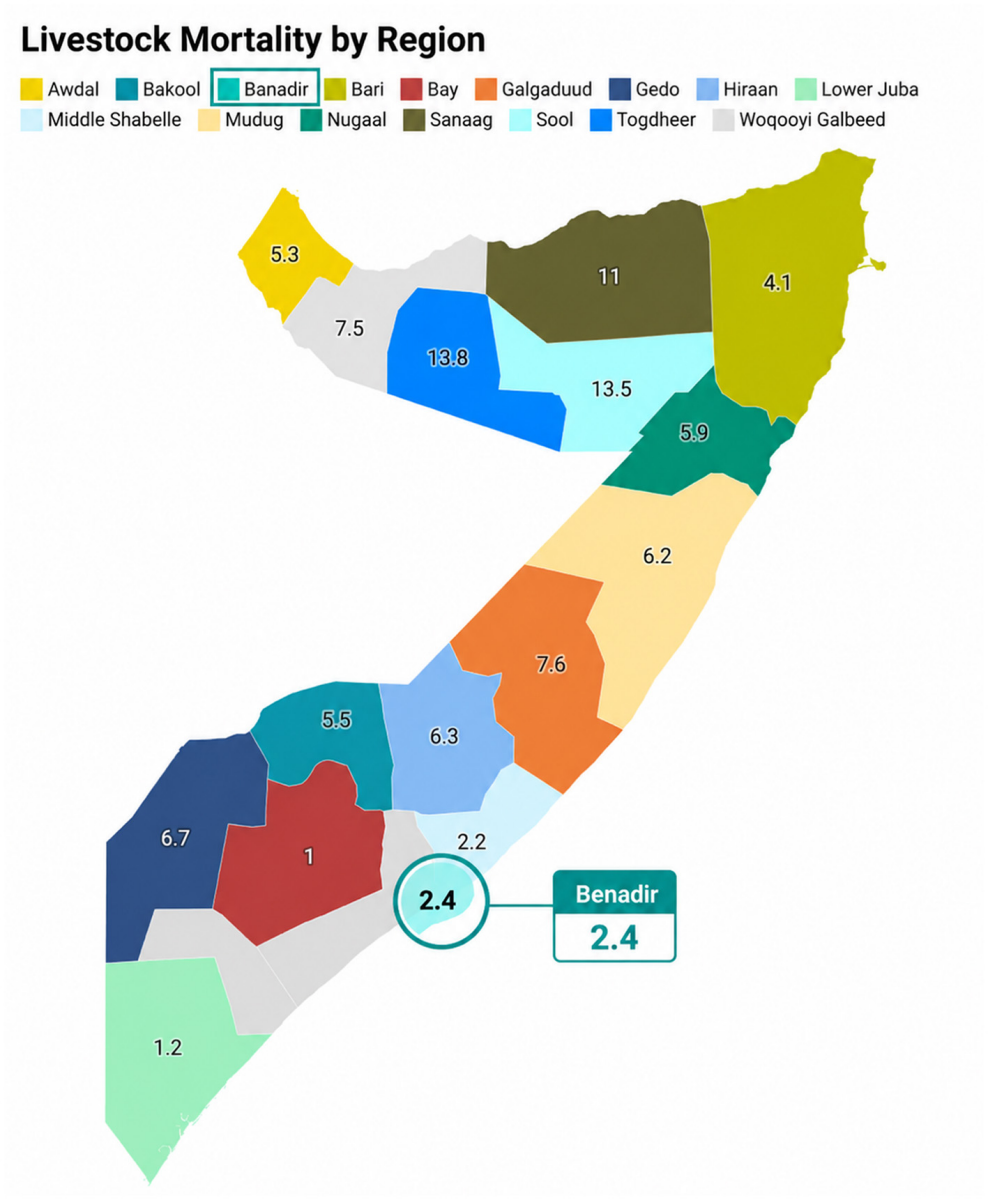
Livestock mortality due to drought and disease by region, Somalia (%).

Drought and disease represent primary threats to livestock survival in Somalia, with devastating consequences for household food security (11). Recent droughts have resulted in massive livestock losses, with some areas experiencing up to 60% herd mortality (12). The 2016–2017 drought alone decreased national livestock numbers from 52.9 million to 36.1 million animals, representing a 32% decline (13). Similarly, livestock diseases contribute substantially to mortality, particularly during and immediately following drought periods, when animals are weakened and more susceptible to infection. These dual shocks create a vicious cycle: livestock deaths reduce household income and food access, while simultaneously eliminating the productive assets needed for recovery (14). The relationship between livestock mortality and food insecurity occurs via multiple pathways. Direct effects include reduced access to animal-sourced foods, such as milk and meat, which are critical for household nutrition. Indirect effects include decreased income from livestock sales, reduced purchasing power for cereals and other foods, and loss of assets that serve as collateral for credit or emergency sales. The cumulative impact extends beyond individual households, affecting local markets through a reduced supply and increased prices of livestock products (12). Despite the recognized importance of livestock in Somali livelihoods, empirical evidence linking livestock mortality with food insecurity at the national level remains limited. Although case studies and regional assessments have documented the impacts of specific droughts or disease outbreaks, comprehensive analyses using nationally representative data are scarce (12). This gap constrains evidence-based policy development and limits our understanding of how livestock mortality patterns vary across different regions, as shown in Figure 1, livelihood systems, and socioeconomic groups (15). This study examined the association between livestock mortality (due to drought and disease) and household food insecurity in Somalia using nationally representative Demographic and Health Survey (DHS) data.

## Methodology

### Study design and data source

This study employed a cross-sectional design using secondary data from the 2020 Somalia Health and Demographic Survey (SHDS). The SHDS is the first nationally representative Demographic and Health Survey conducted in Somalia, implemented between 2018 and 2019 by the Federal Government of Somalia in partnership with the Federal Member States. The survey was designed to provide comprehensive information on population characteristics, health indicators, and socioeconomic conditions across all regions of Somalia. The SHDS utilizes standardized DHS methodologies to ensure data quality and international comparability (16).

### Study population and sampling strategy

The analysis included 26,389 households from 538 enumeration areas (220 urban, 147 rural, and 171 nomadic clusters) across Somalia that provided complete information on livestock ownership, mortality, and food security indicators. The SHDS employed a stratified three-stage cluster sampling design to ensure national representativeness across the urban, rural, and nomadic populations. The sampling frame was constructed separately for the urban and rural areas using available administrative structures, whereas the nomadic populations required specialized enumeration techniques, including nomadic link workers (NLWs) for community mobilization, GPS-based tracking of temporary nomadic settlements (TNS), mobile survey teams with satellite imagery support, and community informants to identify nomadic encampments to account for their mobility patterns. This comprehensive approach ensures the representation of Somalia's diverse livelihood systems and geographic regions (16).

### Outcome variable

Household food insecurity was assessed using the SHDS household questionnaire question: ‘In the past 4 weeks, was there a time when your household did not have enough food to eat?' Households responding ‘yes' were classified as food insecure (coded as 1), while those responding ‘no' were classified as food secure (coded as 0). This binary measure reflects the food access dimension of food security over a 4-week recall period, specifically capturing households' self-reported recent experiences of food deprivation due to insufficient resources. Although this simplified single-item indicator does not assess severity, duration, or other food security dimensions (availability, utilization, stability) captured by multi-item validated scales, it provides a population-level estimate of food access vulnerability (17). It is important to note that this measure represents food access vulnerability rather than a comprehensive assessment of all food insecurity dimensions.

### Explanatory variables

Socio-demographic factors included region of residence, categorized across Somalia's 16 administrative regions; type of residence, classified as urban, rural, or nomadic to reflect different livelihood systems; sex of household head, dichotomized as male or female; and wealth quintile, representing household economic status from lowest to highest quintiles. The wealth quintiles were derived from the national-level SHDS wealth index; however, because this analysis specifically focuses on livestock-owning households, who are disproportionately represented in the lower economic strata compared to the general national population—the distribution across the five categories is not uniform.

Livestock mortality variables captured deaths due to both drought and disease across the four species categories: camels, cattle, small ruminants (sheep and goats combined), and poultry. Drought-related mortality was assessed for these four species categories, with each variable coded as a binary. Specifically, livestock mortality was assessed as a binary variable for each species (0 = no deaths reported or species not owned, 1 = at least one death reported). This household-level indicator captures the occurrence of mortality within the broader population but does not quantify herd-specific mortality rates or account for variations in herd sizes across households. Similarly, disease-related mortality was measured for the same four livestock categories using identical coding. This approach reflects the dual nature of livestock mortality risks in pastoral systems and allows for a population-level examination of species-specific vulnerabilities.

The SHDS assessed mortality due to drought and disease but did not capture losses from wildlife predation or other causes. Although predation may contribute to livestock losses in some pastoral settings, disease and drought represent the primary documented threats in Somalia.

### Livestock ownership variables

Livestock ownership was assessed for four species categories: camels, cattle, small ruminants (sheep and goats, locally termed ‘shoats'), and poultry. For this analysis, ownership was defined as the presence of at least one animal of a given species within the household. These variables were used to characterize the study population and to ensure the species-specific mortality assessments aligned with the primary livestock types managed in Somali pastoral and agropastoral systems.

Food insecurity status was assessed based on a 4-week recall period (past 4 weeks), while livestock mortality data referred to the 12-month period preceding the survey (conducted in 2018 and 2019). Respondents were asked to report whether at least one animal from each species category had died due to drought or disease during the 12-month recall period for livestock mortality.

Household respondents were asked to attribute livestock deaths to either drought or disease based on their direct observations and experiences. While we acknowledge that drought and disease are often epidemiologically and clinically interlinked—as drought-stressed and malnourished animals become immunocompromised and more susceptible to infectious diseases—we report them as distinct categories as recorded in the SHDS questionnaire. No clinical diagnostic data, veterinary confirmation, or detailed symptomatology were collected in the survey; mortality attribution relied entirely on respondent perception. This limitation means that the distinction between ‘drought-related' and ‘disease-related' mortality may not represent true etiological differences but rather household-level interpretations of primary observable causes.

### Statistical analysis

Descriptive statistics were computed to characterize the study population and livestock mortality patterns, with results presented as frequencies and percentages for categorical variables. Univariable logistic regression analyses were conducted to estimate unadjusted odds ratios (OR) with 95% confidence intervals (CIs) to examine the association between each explanatory variable and household food insecurity. Subsequently, multivariable logistic regression was performed, including all predictor variables simultaneously, to estimate adjusted odds ratios (aOR) with 95% confidence intervals. This model adjusted for key structural sociodemographic factors including region, residence type, sex of the household head, and household wealth which were selected as primary determinants of food security and mortality risk in the Somali context.

Model diagnostics were performed to assess stability and fit, including the evaluation of multi-collinearity using variance inflation factors (VIF), which remained within acceptable limits (VIF < 2.0 for all predictors). Goodness-of-fit was assessed to ensure the multivariable model appropriately captured the variation in the data. The multivariable model allowed for the assessment of independent associations between livestock mortality and food insecurity while accounting for fundamental sociodemographic characteristics. Statistical significance was set at *p* < 0.05 for all analyses. All analyses incorporated appropriate survey weights to account for the complex sampling design and ensure population-level inferences. Standard errors were adjusted for clustering at the primary sampling unit level. All sociodemographic and livestock mortality variables were included in the multivariable model regardless of univariable significance levels to control for potential confounding factors.

All statistical analyses, including data extraction and management, were performed using Stata version 17.0 (StataCorp LLC, College Station, TX, USA). Survey weights provided by the DHS Program were applied using the svyset and svy commands.

### Ethical considerations

This study utilized publicly available anonymized datasets that did not contain personally identifiable information. Ethical approval for the original survey was obtained from the DHS Program and relevant Somali authorities, including the Federal Government of Somalia and the Federal Member States. Survey implementation followed established ethical standards, including informed consent and data confidentiality protocols. As this study involved a secondary analysis of de-identified data, no additional ethical clearance was required.

## Results

## Descriptive characteristics

Prior to examining mortality, livestock ownership patterns were characterized by the study population. Ownership was assessed for four species categories: camels, cattle, small ruminants (sheep and goats, locally termed ‘shoats'), and poultry. Of the 26,389 households analyzed, 38.6% (*n* = 10,186) owned at least one livestock species (16). Among all households, 35.8% (*n* = 9,447) owned small ruminants, making them the most owned species, followed by camels at 24.3% (*n* = 6,412), cattle at 11.7% (*n* = 3,087), and poultry at 10.2% (*n* = 2,691). The analysis utilized the full sample of 26,389 households to examine the population-level associations between species-specific mortality and food insecurity.

Table 1a presents the descriptive characteristics of the total analytical sample of 26,389 Somali households and their experiences with livestock mortality. Of these, nearly half (45.7%) reported the occurrence of at least one camel death due to drought, while cattle deaths due to drought were reported by 16.4% of households. Drought-related mortality occurrence was highest for small ruminants, with 91.3% of households reporting at least one death; notably, this high percentage relative to the current ownership rate (35.8%) suggests widespread herd depletion during the 12-month recall period. Regarding disease-related mortality occurrence, 22.3% of households experienced small ruminant deaths, followed by camels (8.6%), poultry (5.0%), and cattle (5.1%). The sample was proportionally distributed across regions, with the largest proportions drawn from Togdheer (13.8%) and Sool (13.5%), while Bay and Lower Juba each contributed less than 2%. More than half of the households were nomadic (52.2%), over two-thirds (69.8%) were male-headed, and three-fifths (61.9%) belonged to the lowest wealth quintile. This distribution reflects the significant concentration of livestock-owning households within the lower wealth strata of the national population (Tables 1a, b). Overall, 42.9% of households reported food insecurity, indicating that nearly half of the surveyed population faced challenges in securing sufficient food.

**TABLE 1 (a):** Livestock mortality due to drought and disease by species (*N* = 26,389).

Variable	Category	Frequency (*n*)	Percent (%)
Camel deaths due to drought	None	14,252	54.3
	≥1 death	11,993	45.7
Cattle deaths due to drought	None	21,931	83.6
	≥1 death	4,313	16.4
Small ruminants deaths due to drought	None	2,282	8.7
	≥1 death	23,918	91.3
Poultry deaths due to drought	None	25,025	95.3
	≥1 death	1,239	4.7
Camel deaths due to disease	None	23,982	91.4
	≥1 death	2,264	8.6
Cattle deaths due to disease	None	24,918	94.9
	≥1 death	1,347	5.1
Small ruminants deaths due to disease	None	20,416	77.7
	≥1 death	5,873	22.3
Poultry deaths due to disease	None	24,946	95.0
	≥1 death	1,308	5.0

**TABLE 1 (b):** Socio-demographic and socio-economic characteristics of households (*N* = 26,389).

Region of residence	Awdal	1,390	5.3
	Woqooyi Galbeed	1,989	7.5
	Togdheer	3,639	13.8
	Sool	3,553	13.5
	Sanaag	2,910	11.0
	Bari	1,074	4.1
	Nugaal	1,550	5.9
	Mudug	1,639	6.2
	Galgaduud	2,001	7.6
	Hiraan	1,664	6.3
	Middle Shabelle	574	2.2
	Banadir	620	2.4
	Bay	266	1.0
	Bakool	1,457	5.5
	Gedo	1,757	6.7
	Lower Juba	306	1.2
Type of residence	Urban	5,037	19.1
	Rural	7,585	28.7
	Nomadic	13,767	52.2
Sex of household head	Male	18,424	69.8
	Female	7,965	30.2
Wealth quintile	Lowest	16,329	61.9
	Second	4,156	15.8
	Middle	3,203	12.1
	Fourth	1,760	6.7
	Highest	941	3.6
Food insecurity (not enough food)	Yes	11,330	42.9
	No	15,059	57.1

Wealth quintiles are based on the national SHDS wealth index. The concentration of the study population in the ‘Lowest' quintile (61.9%) reflects the higher prevalence of poverty among livestock-owning and nomadic households relative to the national average.

## Univariable and multivariable associations

The distribution of food insecurity by sociodemographic characteristics and livestock mortality is summarized in Tables 2, 3. Regional disparities were exceptionally pronounced; while the Bakool (aOR = 14.93), Gedo (aOR = 17.75), and Hiraan (aOR = 10.09) regions exhibited the greatest risk compared to the Awdal (reference), Woqooyi Galbeed (aOR = 1.34), and Lower Juba (aOR = 1.60) regions, which showed substantially lower risk. The magnitude of these associations reflects extreme geographic stratification of food insecurity, though the very high odds ratios in these specific regions should be interpreted as indicators of severe localized vulnerability. Households in rural (aOR = 0.76, 95% CI: 0.69–0.84) and nomadic (aOR = 0.52, 95% CI: 0.46–0.57) settings were associated with significantly lower odds of food insecurity than their urban counterparts. Female-headed households were more likely to experience food insecurity than male-headed households were (aOR = 1.20, 95% CI: 1.13–1.27). Wealth gradients were evident as households in the middle (aOR = 0.72, 95% CI: 0.65–0.80), fourth (aOR = 0.43, 95% CI: 0.38–0.49), and highest quintiles (aOR = 0.28, 95% CI: 0.23–0.33) had significantly reduced odds compared to those in the lowest quintile.

**Table 2:** Univariable regression analysis of factors associated with household food insecurity, Somalia, 2020.

Predictor	Category	Food secure (*n*)	Food insecure (*n*)	Total (*n*)	Unadjusted OR (95% CI)	*p*-value
Region (ref: Awdal)	Woqooyi Galbeed	1,591	398	1,989	1.26 (1.05–1.50)	0.013
	Togdheer	2,514	1,125	3,639	2.25 (1.92–2.63)	< 0.001
	Sool	2,389	1,164	3,553	2.44 (2.09–2.86)	< 0.001
	Sanaag	1,981	929	2,910	2.35 (2.00–2.76)	< 0.001
	Bari	589	485	1,074	4.13 (3.43–4.97)	< 0.001
	Nugaal	1,147	403	1,550	1.76 (1.47–2.11)	< 0.001
	Mudug	882	757	1,639	4.31 (3.63–5.11)	< 0.001
	Galgaduud	827	1,174	2,001	7.12 (6.03–8.42)	< 0.001
	Hiraan	508	1,156	1,664	11.42 (9.58–13.61)	< 0.001
	Middle Shabelle	348	226	574	3.26 (2.62–4.06)	< 0.001
	Banadir	266	354	620	6.68 (5.40–8.26)	< 0.001
	Bay	57	209	266	18.40 (13.29–25.47)	< 0.001
	Bakool	259	1,198	1,457	23.21 (19.10–28.20)	< 0.001
	Gedo	326	1,431	1,757	22.02 (18.29–26.51)	< 0.001
	Lower Juba	216	90	306	2.09 (1.57–2.78)	< 0.001
Residence (ref: urban)	Rural	4,217	3,368	7,585	0.68 (0.63–0.73)	< 0.001
	Nomadic	8,530	5,237	13,767	0.52 (0.49–0.56)	< 0.001
Sex of household head (ref: male)	Female	4,286	3,679	7,965	1.21 (1.15–1.27)	< 0.001
Wealth quintile (ref: lowest)	Second	1,829	2,327	4,156	1.82 (1.70–1.95)	< 0.001
	Middle	1,721	1,482	3,203	1.23 (1.14–1.33)	< 0.001
	Fourth	1,164	596	1,760	0.73 (0.66–0.81)	< 0.001
	Highest	732	209	941	0.41 (0.35–0.48)	< 0.001
Livestock deaths due to drought	Camel	7,361	4,632	11,993	0.72 (0.69–0.76)	< 0.001
	Cattle	1,675	2,638	4,313	2.42 (2.26–2.59)	< 0.001
	Small Ruminants	13,628	10,290	23,918	0.98 (0.90–1.07)	0.679
	Poultry	361	878	1,239	3.42 (3.01–3.87)	< 0.001
Livestock deaths due to disease	Camel	1,170	1,094	2,264	1.27 (1.16–1.38)	< 0.001
	Cattle	406	941	1,347	3.27 (2.90–3.68)	< 0.001
	Small Ruminants	2,693	3,180	5,873	1.79 (1.69–1.90)	< 0.001
	Poultry	283	1,025	1,308	5.19 (4.54–5.93)	< 0.001

**Table 3:** Multivariable regression analysis of factors associated with household food insecurity, Somalia, 2020.

Predictor	Category	Food secure (*n*)	Food insecure (*n*)	Total (*n*)	Adjusted OR (95% CI)	*p*-value
Region (ref: Awdal)	Woqooyi Galbeed	1,591	398	1,989	1.34 (1.11–1.61)	0.002
	Togdheer	2,514	1,125	3,639	2.48 (2.10–2.92)	< 0.001
	Sool	2,389	1,164	3,553	2.63 (2.23–3.10)	< 0.001
	Sanaag	1,981	929	2,910	2.80 (2.36–3.31)	< 0.001
	Bari	589	485	1,074	4.90 (4.04–5.95)	< 0.001
	Nugaal	1,147	403	1,550	2.17 (1.79–2.62)	< 0.001
	Mudug	882	757	1,639	4.86 (4.07–5.81)	< 0.001
	Galgaduud	827	1,174	2,001	7.33 (6.16–8.71)	< 0.001
	Hiraan	508	1,156	1,664	10.09 (8.42–12.09)	< 0.001
	Middle Shabelle	348	226	574	3.02 (2.38–3.83)	< 0.001
	Banadir	266	354	620	4.45 (3.50–5.67)	< 0.001
	Bay	57	209	266	9.45 (6.66–13.41)	< 0.001
	Bakool	259	1,198	1,457	14.93 (12.17–18.33)	< 0.001
	Gedo	326	1,431	1,757	17.75 (14.67–21.49)	< 0.001
	Lower Juba	216	90	306	1.60 (1.19–2.14)	0.002
Residence (ref: urban)	Rural	4,217	3,368	7,585	0.76 (0.69–0.84)	< 0.001
	Nomadic	8,530	5,237	13,767	0.52 (0.46–0.57)	< 0.001
Sex of household head (ref: male)	Female	4,286	3,679	7,965	1.20 (1.13–1.27)	< 0.001
Wealth quintile (ref: lowest)	Second	1,829	2,327	4,156	1.06 (0.96–1.16)	0.245
	Middle	1,721	1,482	3,203	0.72 (0.65–0.80)	< 0.001
	Fourth	1,164	596	1,760	0.43 (0.38–0.49)	< 0.001
	Highest	732	209	941	0.28 (0.23–0.33)	< 0.001
Livestock deaths due to drought	Camel	7,361	4,632	11,993	1.01 (0.95–1.08)	0.733
	Cattle	1,675	2,638	4,313	1.32 (1.21–1.45)	< 0.001
	Small Ruminants	13,628	10,290	23,918	1.11 (0.99–1.23)	0.067
	Poultry	361	878	1,239	1.27 (1.09–1.49)	0.003
Livestock deaths due to disease	Camel	1,170	1,094	2,264	1.12 (1.00–1.25)	0.047
	Cattle	406	941	1,347	1.13 (0.97–1.31)	0.129
	Small Ruminants	2,693	3,180	5,873	1.26 (1.17–1.36)	< 0.001
	Poultry	283	1,025	1,308	1.71 (1.45–2.02)	< 0.001

The finding that rural and nomadic households were significantly less food-insecure than their urban counterparts warrants further investigation, as it contradicts common assumptions that urban residents benefit from better market access and non-agricultural employment opportunities. This counterintuitive result may reflect several mechanisms: urban households‘ greater vulnerability during supply chain disruptions and food price inflation, particularly when reliant on purchased foods; the higher levels of food self-sufficiency in rural settings, where households often produce their own food staples locally and are therefore less vulnerable to market price shocks; the protective buffering capacity of livestock assets in pastoral systems, which provide direct access to milk and meat during shortages; and the social safety nets and reciprocal exchange systems more prevalent in rural and nomadic communities. Additionally, nomadic populations' mobility may enable flexible migration to areas with better resource availability during crises, while urban residents face fixed location constraints.

In terms of livestock mortality, adjusted models showed that household food insecurity was significantly associated with cattle deaths due to drought (aOR = 1.32, 95% CI: 1.21–1.45) and poultry deaths due to drought (aOR = 1.27, 95% CI: 1.09–1.49). Disease-related mortality demonstrated comparable or stronger associations: small ruminant deaths (aOR = 1.26, 95% CI: 1.17–1.36) and poultry deaths (aOR = 1.71, 95% CI: 1.45–2.02) were both significantly associated with higher odds of household food insecurity. Conversely, camel mortality due to drought was not significantly associated with food insecurity after adjustment, while camel mortality due to disease showed a marginal positive association (aOR = 1.12, *p* = 0.047) (Table 3).

## Discussion

This study provides nationally representative evidence on the relationship between livestock mortality and household food insecurity in Somalia using recent Demographic and Health Survey data. The analysis revealed a high prevalence of food insecurity, affecting 42.9% of households, with substantial variation by region, sociodemographic characteristics, and patterns of livestock deaths. Adjusted regression models indicated particularly elevated odds of food insecurity in the Bakool, Gedo, and Hiraan regions; female-headed households faced an increased risk, while rural and nomadic households demonstrated protective effects; higher wealth quintiles exerted a marked protective effect. Livestock mortality was significantly associated with food insecurity, particularly deaths of poultry and small ruminants due to disease, which remained strongly associated with heightened food insecurity in the adjusted analyses.

The association between livestock mortality and food insecurity aligns with the finding that disease-related poultry mortality was associated with 71% higher odds of food insecurity. This observation is consistent with recent assessments of food security in Somalia and the wider Horn of Africa, where acute food insecurity, malnutrition, and vulnerability to climatic shocks remain endemic (18). Previous studies have highlighted the centrality of livestock as both a livelihood and nutritional anchor in Somali, Ethiopian, and Kenyan drylands (19). The observed association between poultry and small ruminant mortality and household food insecurity supports earlier research indicating that small livestock provide easily accessible dietary protein and income and are crucial for resilience during droughts or market shocks (20). Disease outbreaks, such as Rift Valley Fever and Peste des Petits Ruminants (PPR), have been documented to disproportionately affect small ruminants and poultry, posing a significant risk to food security for pastoral households in the region (21). Notably, the finding that rural and nomadic residents were relatively protected contrasts with some literature suggesting that these groups are highly vulnerable; however, it aligns with evidence that mobility and diversified herd management can buffer shocks by enabling flexible coping strategies (22).

As noted above, marked regional disparities were observed, with some regions showing exceptionally high odds ratios (Bakool: aOR = 14.93; Gedo: aOR = 17.75), while these large estimates may be sensitive to sparse-data bias or localized model instability, they reflect the convergence of ongoing humanitarian crises, recurrent droughts, and conflict-induced displacement documented in these localities during the survey period. Differences between urban and nomadic households may arise from mobility-driven coping mechanisms; despite their exposure to environmental hazards, nomadic populations often deploy flexible migration, herd diversification, and social networks to mitigate food shortages (19). Additionally, rural and nomadic households often benefit from higher levels of food self-sufficiency through local production and direct access to animal-source foods, making them less vulnerable to market price shocks. In contrast, urban households, especially those reliant on market purchases, are more exposed to food price inflation and less able to absorb shocks from livestock and crop losses. The increased likelihood of food insecurity among female-headed households corresponds with broader evidence from sub-Saharan Africa that gendered barriers to accessing resources, social capital, and income may exacerbate vulnerability (22). Finally, the strong gradient observed across wealth quintiles—which reflects the significant concentration of livestock-owning households within the lower economic strata of the national population is consistent with research emphasizing economic status as a fundamental determinant of food security, shaping the ability to recover from shocks and access public and private resources (18).

Deaths of poultry and small ruminants, especially those attributed to disease, were most strongly and consistently associated with household food insecurity, even after accounting for sociodemographic factors. This pattern likely reflects the rapid and direct contribution of these species to daily food consumption and income; poultry and small ruminants are more frequently sold for cash or slaughtered for household use, while also being more susceptible to episodic disease outbreaks, such as Newcastle disease (poultry) or PPR (small ruminants) (23). These results align with intervention studies showing that vaccination and veterinary support for small livestock can have substantial positive effects on household nutrition and economic security (18). The attenuation in associations observed for camel and cattle mortality occurrence after adjustment may reflect species-specific resilience; camels and cattle, although economically significant, are less frequently turned over for immediate household consumption and often represent longer-term capital that buffers households over time. Previous studies in Somalia have noted a relatively lower short-term impact on household dietary diversity following large animal losses compared with small stocks (19).

## Policy and practical implications

These findings underscore the importance of prioritizing livestock health interventions within Somali food security and resilience strategies. Vaccination campaigns for poultry and small ruminants, improved veterinary care, and targeted disease surveillance are likely to yield significant gains in food security (24). Drought preparedness measures, such as fodder assistance, supplemental feeding (e.g., rangeland cubes), water infrastructure, and restocking initiatives, should be integrated into a One Health approach that addresses the intersection of livestock, humans, and environmental health (22). Tailoring interventions to regional needs and recognizing protective factors among nomadic populations (e.g., mobility and network-based social support) will strengthen adaptation planning and improve humanitarian targeting (19). This evidence can inform Somali national adaptation plans and multisectoral food security efforts, emphasizing the need for sustained investment in both livestock support and gender-sensitive social protection (18).

## Strengths and limitations

This study has several limitations. The cross-sectional design precludes causal inference; as livestock mortality and food insecurity were assessed simultaneously, these findings should be interpreted as associations rather than causal effects. Importantly, livestock mortality was measured as binary household-level occurrence data (≥1 death) rather than biological mortality rates. Without data on initial herd sizes, the analysis could not determine the specific proportion of animal loss within individual households. Furthermore, the study utilized the full household sample (*N* = 26,389) to examine population-level associations; while this provides a broad overview, including households that do not own specific species in the reference group may influence the magnitude of some associations.

Despite controlling key sociodemographic factors, residual confounding is likely substantial. Due to survey constraints, we were unable to adjust for several important determinants of both livestock mortality and food insecurity, including household size, educational attainment, initial herd size, internal displacement status, and direct conflict exposure. Additionally, factors such as water access and livelihood diversification were not included in the multivariable models. The use of a national-level wealth index also resulted in a skewed distribution, as livestock owners are disproportionately represented in the lower national economic strata. Furthermore, the exceptionally high adjusted odds ratios reported for specific regions should be interpreted with caution; while they reflect severe regional disparities, such large estimates in multivariable models can be sensitive to sparse-data bias or localized model instability. Mortality attribution relied on respondent perception without clinical verification. Finally, the temporal mismatch between the 12-month mortality recall and the 4-week food insecurity recall means we cannot account for intervening shocks. Despite these limitations, this study provides the first nationally representative evidence of livestock mortality food insecurity associations in Somalia using a robust multivariable analysis.”

## Conclusion

This study demonstrates a strong association between livestock mortality occurrence, particularly among poultry and small ruminants due to disease, and household food insecurity in Somalia. These findings highlight the urgent priorities for targeted livestock health interventions, drought adaptation, and One Health programs as part of broader food security strategies. Notably, rural and nomadic households demonstrated lower food insecurity than their urban counterparts, likely reflecting the protective buffering capacity of livestock assets and higher levels of food self-sufficiency in rural production systems. Future research should investigate these dynamics further using longitudinal designs and a broader range of covariates, including conflict dynamics, displacement history, and herd-specific metrics to further isolate the impact of livestock-related shocks on household resilience in pastoral and agropastoral contexts.

## Data Availability

The datasets presented in this study can be found in online repositories. The names of the repository/repositories and accession number(s) can be found below: https://microdata.nbs.gov.so/index.php/catalog/50.
